# A Checklist for Implementing Rural Pathways to Train, Develop and Support Health Workers in Low and Middle-Income Countries

**DOI:** 10.3389/fmed.2020.594728

**Published:** 2020-11-27

**Authors:** Belinda O'Sullivan, Bruce Chater, Amie Bingham, John Wynn-Jones, Ian Couper, Nagwa Nashat Hegazy, Raman Kumar, Henry Lawson, Viviana Martinez-Bianchi, Sankha Randenikumara, James Rourke, Sarah Strasser, Paul Worley

**Affiliations:** ^1^Faculty of Medicine, Rural Clinical School, University of Queensland, Toowoomba, QLD, Australia; ^2^Faculty of Medicine, Rural Clinical School, University of Queensland, Theodore, QLD, Australia; ^3^Keele Medical School, Keele University, Keele, United Kingdom; ^4^Ukwanda Center for Rural Health, Department of Global Health, Stellenbosch University, Cape Town, South Africa; ^5^Medical Education and Human Resources Center, Faculty of Medicine, Menoufia University, Shibin el Kom, Egypt; ^6^Family Medicine Practitioner, DOC24 Family Practice Clinic, Ghaziabad, India; ^7^Ghana College of Physicians and Surgeons, Accra, Ghana; ^8^Department of Family Medicine and Community Health, Duke University, Durham, NC, United States; ^9^Planning and Quality Management Unit, Base Hospital, Panadura, Sri Lanka; ^10^Center for Rural Health Studies, Memorial University of Newfoundland, St. John's, NL, Canada; ^11^College of Medicine and Public Health, Flinders University, Adelaide, SA, Australia

**Keywords:** rural workforce, rural health workers, training, education, professional support, implement, LMICs, guide

## Abstract

**Background:** There is an urgent need to scale up global action on rural workforce development. This World Health Organization-sponsored research aimed to develop a Rural Pathways Checklist. Its purpose was to guide the practical implementation of rural workforce training, development, and support strategies in low and middle-income countries (LMICs). It was intended for any LMICs, stakeholder, health worker, context, or health problem.

**Method:** Multi-methods involved: (1) focus group concept testing; (2) a policy analysis; (3) a scoping review of LMIC literature; (4) consultation with a global Expert Reference Group and; (5) field-testing over an 18-month period.

**Results:** The Checklist included eight actions for implementing rural pathways in LMICs: establishing community needs; policies and partners; exploring existing workers and scope; selecting health workers; education and training; working conditions for recruitment and retention; accreditation and recognition of workers; professional support/up-skilling and; monitoring and evaluation. For each action, a summary of LMICs-specific evidence and prompts was developed to stimulate reflection and learning. To support implementation, rural pathways exemplars from different WHO regions were also compiled. Field-testing showed the Checklist is fit for purpose to guide holistic planning and benchmarking of rural pathways, irrespective of LMICs, stakeholder, or health worker type.

**Conclusion:** The Rural Pathways Checklist provides an agreed global conceptual framework for the practical implementation of “grow your own” strategies in LMICs. It can be applied to scale-up activity for rural workforce training and development in LMICs, where health workers are most limited and health needs are greatest.

## Introduction

Poor access to healthcare has major implications for the health and well-being of millions of people. Globally, countries with the highest proportion of rural residents correlate with poorest access. In countries where rural populations exceed 70%, only 16% of the population has access to universal health coverage (1). The most affected people are those in rural communities of LMICs (2). More than 90 per cent of people of low-income countries have no access to basic health care (3).

A critical issue for increasing universal health coverage is addressing the availability of a skilled rural health workforce (4, 5). The International Labor Organization (ILO) estimates that there is a current global shortfall of 10.3 million health workers needed to ensure provision of quality health services (3). Further, the World Health Organization (WHO) suggests that, based on increasing healthcare demand, ~18 million health workers are needed by 2030, mainly in LMIC (6).

To increase access to rural and remote health workers worldwide, the WHO released its evidence-based global recommendations of effective strategies in 2010 (7). These focus on four areas of intervention: rural education, regulatory, financial, and personal and professional support. Of these strategies, investing in “grow your own” educational approaches (selecting and training health workers in rural areas) are considered as a mainstay. They particularly play a role in achieving a skilled and satisfied rural workforce, over relying on regulatory strategies like obligatory rural service requirements (8–14). However, educational strategies are some of the most complicated to translate into practice because they involve multiple stakeholders and complex actions at different systems levels. Compared with financial or regulatory strategies which can be enacted through a change in central government policy or legislation, to be successful, rural educational strategies involve a holistic package of linked interventions that are managed over a longer-term cycle. Further, they rely on tailoring to different rural places, training systems, types of health workers, and health system infrastructure. This makes their implementation relatively complicated, particularly for LMICs where there are many competing healthcare priorities to address within restricted resources.

Effective translation of the WHO recommendations into practice is also challenging because there are no known practical tools which integrate the WHO “rural education” and “personal and professional support” strategies into a package for implementation. This is important as rural education, unless backed up with ongoing development and support, is likely to fail as a longer-term workforce development strategy. The availability of practical tools like Checklists, may help to integrate rural training and professional development and support actions, tailored to different LMICs and rural and remote settings.

Implementing training and development strategies in rural locations, apart from improving access to healthcare, has a significant role in addressing rural social and economic development. This approach rebalances the range of training and practice resources, skills, and jobs that are typically concentrated in cities, to be available in rural communities. The WHO High-Level Commission identifies the links between health and health sector jobs, in rural communities and social, economic, and health outcomes (15). The urgent need for more health workers in LMICs over the next 15 years presents a significant global challenge, but by addressing this challenge through fostering more rural-based health worker training and development interventions, it is possible also to generate economic growth where it is most needed (15). Developing more rural training and development also reinforces the United Nations' Sustainable Development Goals, by improving access to rural quality education and work, they reduce poverty, improve gender equality, build community partnerships, and promote health and well-being in rural places (16).

There is no globally agreed and LMIC-sensitive terminology that addresses the training, development, and support strategies needed to grow the rural workforce. Colloquially, the term “*grown your own*” and “*rural training*” only picks up on elements related to training. “*Rural pipelines*” is also often used, however, it purveys being stuck in a rigid structure “*pipeline*.” The term “*rural pathways*” has the potential to better reflect the choice of the trainee/worker to participate at each stage of their development and ongoing work, as a continuum of experience toward being skilled and supported for ongoing work in rural communities.

Our research aimed to confirm whether *rural pathways* terminology was appropriate, and describe what it entailed. Secondly, we aimed to design a Rural Pathways Checklist (Checklist) as a tool to guide the implementation of rural pathways in LMICs contexts. Finally, we intended to disseminate and test the application of the Checklist, assessing whether it addressed its purpose.

## Methods

This project was sponsored by the WHO and had ethics approval from Monash University, Victoria, Australia (Project number 17636) ratified by the University of Queensland (Project number 2019002437). At project commencement, we engaged 13 experienced rural pathways implementers/rural health researchers from around the world, in a Steering Committee (October—December 2018). This group had experience of LMIC settings in different WHO regions and oversaw all aspects of project governance, quality, decision-making, and engagement. The Steering Committee, in consultation with the WHO, firstly agreed a clear vision, principles, and methods for the Checklist. These are outlined below.

### Focus Groups

Two focus groups were held face-to-face with LMIC participants at the World Rural Health Conference (run by Rural WONCA—the Working Party on Rural Practice of the World Organization of Family Doctors) in 2018. These aimed to test LMIC concepts and terminology for “*rural pathways*,” “*training*,” “*recruitment*,” and “*professional support*.” Participants were trained primary healthcare workers and rural educators. They participated in open discussion with facilitators, where group comments were noted on a whiteboard, printed out and shared with the research team for analysis and informing the next stages of a policy and scoping review.

### Policy Review

A desktop review of existing LMIC rural health workforce policies aimed to describe the progress and outcomes of rural focused policies and programs in LMIC settings, based on concepts and terminology from the focus groups. This was considered an important background for interpreting rural pathways strategies and describing the context of implementation. Articles or reports were accessed via key websites, sourced by the Steering Committee and Expert Reference Group or identified from published papers about LMIC human resource and rural pathways policies (by-products of the scoping which is described below).

### Scoping Review

A scoping review was done using the five stage Arksey and O'Malley process (17, 18). The research question was to identify the extent, range and nature of LMIC rural pathways evidence and identify any exemplars. The search strategy and inclusion criteria (Table 1) were iteratively developed and informed by the LMICs concepts/terminology emerging from the focus groups, Steering Committee discussions and other global workforce reports (7, 20). In order to identify material and activity occurring in Latin-American and Francophone countries, English, French, and Spanish articles were included.

**Table 1:** Search strategy and inclusion criteria.

**Item**	**Description**
Search period	The search was limited to literature published between 1st January 1998 and 30th October 2018
Databases	Six databases were chosen based on scope and relevance of literature content: Medline, Social Science Citation Index, CINAHL, ERIC, Rural and Remote Health, Informit Health Collection, and the Cochrane Database of Systematic reviews. The search strategy included a Boolean search using the three sets of search terms
Additional sources	Other literature was also identified from snowballing, hand searching and directly identified by the Steering Committee and an Expert Reference Group
Sensitivity	A sensitivity analysis of the search strategy was performed, ensuring that results included known or key texts identified by steering committee members
Search concepts	The concepts applied to the search were based on the review question: *What are the main elements of rural pathways to train and support the rural workforce in LMIC and what are their outcomes? What contextual factors influence implementation of rural pathways in LMIC to inform a Checklist for these countries? Are there any vignettes of best practice models that would support reflection?* Concept 1: Rural or remote. Concept 2: “health work*” or doctor OR “general practitioner” OR “physician OR nurse” OR “nurse practitioner” OR “rural generalist” OR “rural nurse” OR “allied health” OR dentist OR specialist OR “community health worker” OR “family physician” OR “family doctor” OR “health prof*” OR “clinical officer” OR “clinical assistant” OR “health assistant” OR “mid-level worker.” Concept 3 train* OR curricul* OR develop* OR course OR placement OR immersion OR skill OR education OR qualification OR competen* OR recruit* OR retention)
Inclusion criteria	Rural or remote Based in a LMIC (or literature review which incorporated LMICs) (19) About any type of health workers in frontline clinical services (excluding non-clinical or liaison roles) Outcomes of any aspect of rural pathways to develop the capacity, skills, scope or distribution of the rural workforce based on the WHO framework (themes of education and training and professionally supportive environment) (7) 1998–2018 English, Spanish, or French
Exclusion criteria	High-income country consistently over last 20 years No outcomes reported—discussion of an intervention only or broadly about human resource statistics rather than rural pipeline LMIC-based training for developing health workers from high-income countries Technological interventions but not specific to supporting rural workforce in LMIC setting Not about rural pathways for the growth and development of the rural workforce <15 people in sample About worker satisfaction with limited breakdown to inform supportive environment factors About intention for rural practice if not linked to a rural pathways factor (such as broad surveys of University student cohorts without delineating relationship to rural training) Rural training was <3 weeks duration Full text not available (via find full text using Endnote, Google or direct library searching)

### Consultation

Consultation with an Expert Reference Group aimed to test and refine the Checklist with practical users, including stakeholders involved in rural workforce development in LMICs as well as personnel in high income countries (HICs) with knowledge of LMICs rural contexts and training systems. The Expert Reference Group included 70 rural pathways implementers who were part of the global rural health policy and practice community associated with Rural WONCA. Participants covered a range of health disciplines, countries and WHO regions, 42 (60%) were from LMICs. Consultation occurred over three stages, via tailored *Qualtrics* surveys sent by Email using Rural WONCA's regular Email list (covering all countries, rural stakeholders, and health worker types). The first round sought to gather general information about rural pathways activity for informing Checklist development. The 2nd and 3rd rounds sought feedback about the draft Checklist (Table 2).

**Table 2:** Questions posed during Expert Reference Group consultation.

**Round**	**Focus**	**Questions**
1	Provide information about rural pathways activities in your area	What rural pathways strategies using, where, the aim, enablers, barriers?
2	Feedback on a draft copy of the Checklist (with reflections and dot points of individual textual description about cited published evidence per action)	What do you think about the range, quality and structure of the draft Checklist? Who would use the Checklist in your setting? What do you prefer— “pathways” or “pipelines” for describing this activity?
3	Feedback on a graphically-designed copy of the Checklist (with reflections and a brief summary of evidence per action)	Is there anything missing? In which action areas have you already made progress? What would enable implementation of actions and how would you measure success?

### Dissemination, Engagement, and Field-Testing

Dissemination, engagement, and field-testing of the Checklist occurred between January 2019 and February 2020. To aid distribution and access to the materials, a short version of the Checklist which was translated into 12 languages: Arabic, Bengali, Burmese, Chinese, French, Hindi, Indonesian, Portuguese, Russian, Spanish, Swahili, and Thai. The Checklist and translated materials were then provided on the Rural WONCA website and sent by email circulation to the global rural health policy and practice network associated with Rural WONCA and other experts these people identified. The materials were also provided to the Expert Reference Group. The Checklist was then presented at six international primary care conference workshops in 5 locations (Kyoto, Bratislava, Darwin, Uganda, Albuquerque), and at key workforce planning networks that the Steering Committee members were involved in.

The field-testing process followed and aimed to test whether the Checklist addressed its intended purpose, including being applicable to a range of LMICs countries and rural and remote contexts, health workers, stakeholders, and rural health problems, for its planned purpose of benchmarking and planning rural pathways implementation. To do this, interactive workshops were held, inclusive of a mix of stakeholders at conferences. Further, direct meetings were held with Expert Reference Group members from LMICs who nominated an interest in the Checklist and a willingness to test its application to their situation.

To aid data collection from meetings and workshops, a self-assessment tool, which was an adaptation of the Checklist, was applied to field-testing. This was developed by changing the reflective questions of the Checklist action areas into a series of 4–6 statements representing components of the action. A copy of this tool and the Checklist was provided to participants in advance of field-testing workshops or meetings.

For field-testing, the Checklist and its intended purpose was firstly explained. Then individuals were asked to consider a problem in their own rural community and the types of health workers they needed to support it. Following this, participants were asked to apply the self-assessment tool to rate their progress on actions for this issue (from 0 to 4 as “nil” to “strong”). The sum of progress scores on each item was then calculated giving participants an overall benchmark score of their progress in each Checklist action area. The process took ~40 min to complete and was followed by 20 min of discussion and reflection, whereby a facilitator recorded participant feedback. When the session concluded, the completed self-assessment tools were collected as data. In addition to this, participants were encouraged to take a copy of the Checklist and materials and apply them to real-world planning in their own communities and submit more detailed feedback via email.

## Results

The agreed vision for the Checklist was *to inform the implementation of all levels of action required for effectively training and supporting health workers in LMICs to improve the capacity, skills, scope, distribution, and retention of qualified rural generalist teams, adaptable for different rural, and remote communities*. For impact, the focus was not just on the quantity of health workers, but also on their quality and relevance to the community (21).

The Checklist principles were also agreed: the Checklist would be informed by the literature about successful LMICs strategies; easy to use; based on universally understood terminology; able to promote reflection and learning; and applicable for planning tailored action for different LMICs, stakeholders, health workers, and community problems/contexts, at any stage of rural pathways development.

### Focus Groups

From the focus groups, LMICs stakeholders identified that the term “*rural pathways*” covered broad strategies encompassing recruitment, education, training, professional support, and retention. In their context, bridging courses and step-wise qualifications were commonly used. Producing fit for purpose generalist teams of rural health workers was agreed to include people with certified skills. Trained or untrained health workers with only basic general qualifications were not considered to meet the criteria, nor support rural equity, or quality care.

### Policy Review

Over 350 articles or reports were accessed in relation to LMIC policies. Substantial variation of rural workforce policy development was apparent for different LMICs and WHO regions. Few countries had strong national-scale rural health or rural workforce strategies. If they did, the strategic directions were often confined to one health worker type or targeted at a particular community health issue like maternal health (22, 23). The evidence of integrated rural pathways strategies was most developed in Thailand and the Philippines for medical doctors, including at University and early graduate levels (24–28). There were also examples of step-wise models included Community Health Worker training in Ethiopia (29, 30) and international partnerships to boost intelligence and resources for building rural pathways (31). However, there was limited evidence of holistic planning for training and supporting the range of skilled health workers needed by rural communities (32–34). This highlighted the potential value of a Checklist as a systems framework.

### Scoping Review

**Figure 1:**
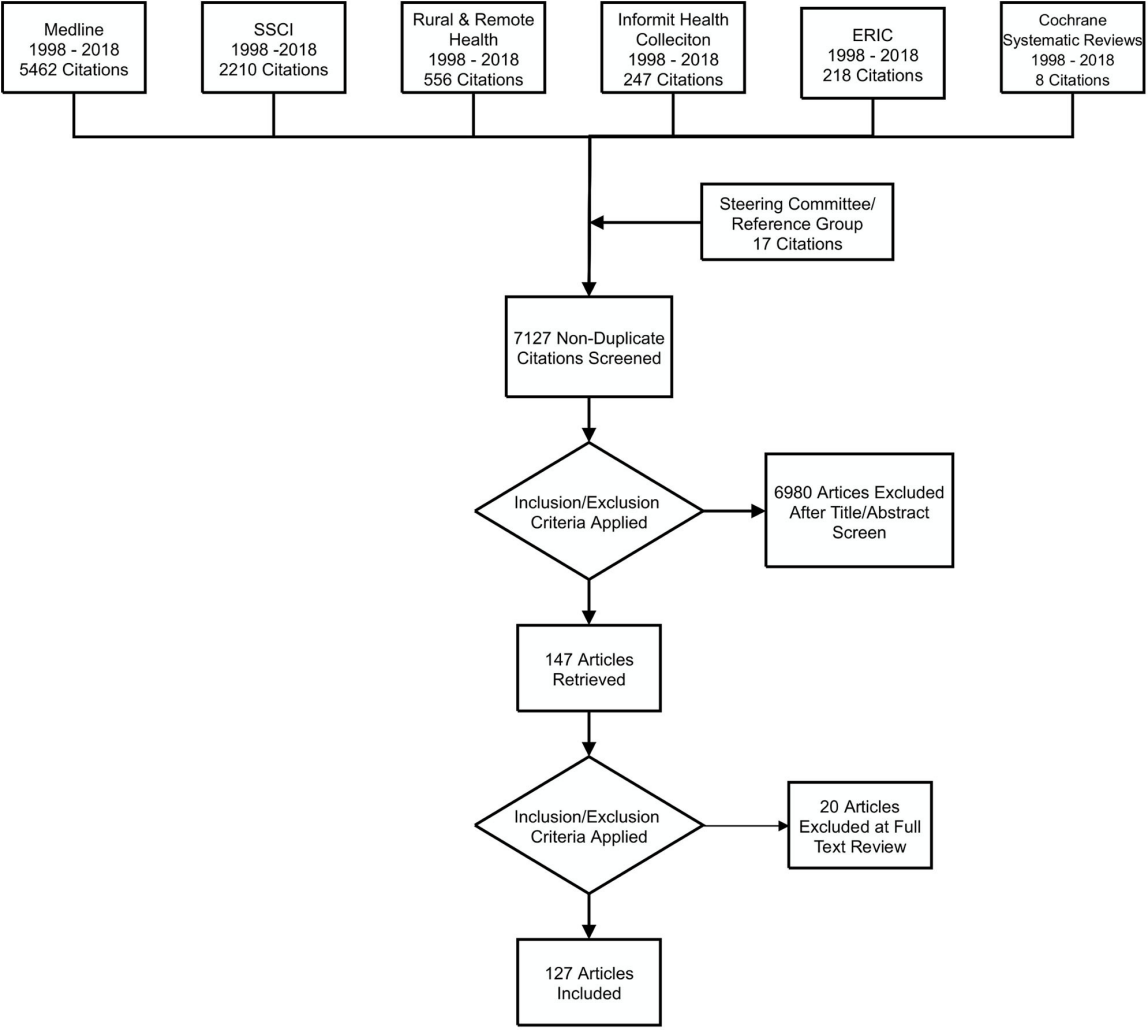
Outcomes of the search strategy.

The scoping review identified 7,127 articles (Figure 1). After screening titles and abstracts, 97 empirical studies (83 from the literature search and 14 identified by the Steering Committee and Expert Reference Group) and 30 literature reviews (27 from the literature search and 3 from the Steering Committee) were included. After full text screening, data extraction was done using an Excel spreadsheet based on criteria that had been iteratively developed to ascertain material relevant to the scoping review question.

Most of the evidence was published since 2010. Empirical studies were from Africa (*n* = 45); Southeast Asia (25); Western Pacific (14); South or Central America (12), and Eastern Mediterranean regions (2) as formally defined WHO regions (35). Of the 97 empirical articles, 63 were about doctors/physicians; 28 were about other primary health care workers, mainly Community Health Workers, Health Extension Workers, Mid-Level Health Workers, Health Assistants, and Auxiliary Midwives. There were also two studies about other disciplines: Radiographers and Nutritionists/Physiotherapists/Speech Therapists/Occupational Therapists. Three other studies focused on Midwives, Nurse Practitioners, and Nurses, respectively. A single study concerned Rural Health Service Managers.

The literature was firstly charted according to broad training and personal/professional support categories based on the strategy areas of the 2010 WHO global recommendations for *Increasing access to health workers in remote and rural areas through improved retention* (7). Inductive thematic analysis was then applied to progressively layer and reorganize material into key themes related to guiding implementation without any pre-existing coding framework. This resulted in a draft framework of rural pathways actions that was discussed and refined based on feedback from the Steering Committee, Expert Reference Group, and staff at the WHO. Eventually the draft Checklist comprised eight equally balanced actions, presented in a Microsoft Word document (shown in Table 3 before being applied as the graphically-designed Checklist in Data Sheet 1). These actions were: establishing community needs, policies, and partners; exploring existing workers and their scope; selecting health workers; education and training; considering working conditions for recruitment and retention; accreditation and recognition of qualified workers; professional support and up-skilling and; monitoring and evaluation. For each action area, reflective questions underpinned by supporting evidence (individual references to research and what it showed) was developed. This design specifically aimed to address the objective of stimulating reflection and learning for implementing each action specific to the LMICs context.

**Table 3:** Rural Pathways Checklist including eight actions^a^.

**Checklist action**	**Summary of evidence**	**Reflective questions**
Community needs, rural policies, and partners	Working with rural communities to explore their needs for healthcare helps to work out priorities for action community. A scan of the national policies and plans for rural health provides insights into directions for governments and potential synergies between policies and the local priorities. Priorities may need to be sorted into an order, particularly in the face of competing demands for resources and in some cases, extensive unmet need. Government and other partners, along with decentralized finance and management is important for enabling solutions to be appropriately tailored and for ensuring appropriate technical and financial support is available.	What do our rural communities need? • Is the community involved in defining priorities and possible solutions? • What are key priorities now, which can be built on later? What rural health policies/plans exist to support action? • Are they implemented? • Do these cover health workforce, training, and rural health priority areas? • Is policy/planning decentralized? • Are new policies needed? What global, national, or local partnerships can we build to help? • Who can assist? • How can the partnership be sustained? (7, 8, 23, 25, 36–43)
Existing workers and their scope	The skill levels of rural workers may not be sufficient to meet rural and remote community needs. A scan of existing rural and remote health workers and their skills, practices, and motivations can inform rural pathways strategies. Rural and remote healthcare teams having a wider range of skills, supported by organizations to address community need, can improve comprehensive local care, and potentially help to improve health worker satisfaction and retention. Communities need to balance short-term recruitment needs with long-term workforce building processes.	What rural healthcare teams, working within what scope, are needed? Do workers already exist with skills for this scope of work, easily recruited/retained? • What are their qualifications and training relative to the skills demands of the role? • Are they motivated to work at the required scope? • Is their health service supporting their increased scope? • Are they being retained in rural and remote areas? • Are they attracted to work in rural and remote communities? • Are there short-term recruitment options whilst longer-term workforce is developed? (41, 44–54)
Selection of health workers	An extensive range of community selection options are demonstrated involving selecting people with a connection to “place,” commitment to serve others, motivated to learn, and invested in improving access to community health services. Universities and training courses with a social accountability for developing health workers with a desire to serve others, trained, and ready to work where they are most needed, tend to select students committed to helping underserved. Selection of rural background, people from disadvantaged communities of different race and language groups relative to the country and rural context is important, along with financial and social support for these groups to fully participate in city courses. Cost benefits of developing new workers are important considerations and should be evaluated.	How can we select workers for this role from the community? • Are there people in the rural community who could fill roles with some education and training? • What process and criteria will effectively select them *from the community for the community*? • What is the entry level standard appropriate for coping with the training? • What financial and social support do community members need to access training? • What are the cost-benefits of training a new worker and who will share the costs of training? (27, 28, 32, 55–70)
Education and training	Optimal education and training for rural practice occurs through exposure to rural and remote practice, teams, and health systems. Learning the range of skills needed is effective through distributed training systems using locally-available qualified teachers and supervisors, in the place where people are going to practice and involving of the people that the workers are going to help after they finish training. This often occurs within University and other training organizations with a social accountability for developing health workers with desire to serve others, trained, and ready to work where they are most needed. And also by providing options for existing rural workers to learn and get qualified, on the job, through supervision, and decentralized courses. For optimal effect, more practical training in the rural communities is best, along with bundled support to optimize the educational experience. Compulsory service strategies work best if they are combined with selection, education, and support strategies. Beyond any one course, there should be options for doing more advanced training, for career progression. Training covers the breadth of skills needed for the role. Sustainable funding and technical support for decentralized training is important.	How can we effectively educate, train, and up-skill people *in* rural areas and for the breadth of skills needed by rural communities or support existing rural workers to learn on the job? • What bridging courses are required? • What rural curriculum is relevant? Who will develop and validate this? • How can theoretical and practical components be delivered in rural areas? • How much real-time supervision and virtual supervision will work to learn practical skills safely? • How can practical learning support the scope and complexity of skills required? • What further training can the qualification articulate with for career development (short course or university)? • How much would it cost to train/employ/support students and how can this be funded? • How can the local government, community, and champions support the training? (8, 24–28, 33, 34, 56, 62, 71–91)
Working conditions for recruitment and retention	Education and training is only likely to be effective in recruiting and retaining health workers if the practice conditions are right, there is a supportive learning culture and strong management in the health service and there are supplies, clinical infrastructure, safe housing, good remuneration, and sustainable workload. Health worker motivation and engagement is better if employers regularly check in with them about their goals and any factors impacting their performance. Structured orientation and community-based projects for new staff can improve transition to rural work as a new worker and interest in continuing in the role.	What are the practice conditions in the community which could affect satisfaction, recruitment and retention? • Are we recruiting people who completed training in the community to work in the community? • Do the rosters make the workload sustainable? • Are we creating jobs with satisfactory employment terms and variety, volume, and scope of work? • Is remuneration appropriately rewarding employees? • Is there an orientation to the workplace? • Is there orientation to the community? • Are senior workers and supervisors available onsite/virtual? • Is there training for health service managers? • What support is there for housing and meals? • Do health workers have transport for their work? • Are there baseline stocks of medical supplies, equipment, and drugs? • Are the health service buildings and clinical infrastructure of reasonable standard? • Is there security for workers? • Are workers given enough time off? • Are there subsidies for work away from home? • Do workers have access to technology support and internet? • Is there rural health team cohesion? (62, 92–103)
Accreditation and recognition	Accreditation and formal professional recognition of the worker is important for recognizing the worker's training and scope of work. It helps reinforce their investment in doing more training and supports their retention in the role and use of all their skills. Clear accreditation and recognition also helps the community to identify qualified health workers. Recognition of supervisors is equally important.	How can people who are trained for rural work be accredited and recognized for transferability of the qualification? • What qualification can they be given? • How can the community value graduates of the training? • Is there a professional title for graduates? • Are the graduates recognized at country level for what they do? • Can the graduates be paid appropriately for using the skills they have developed? • Do they have options for progressing their career path? (62, 81, 92, 96, 104, 105)
Professional support and up-skilling	It is important to provide professional supervision and networking opportunities to reduce health worker isolation and reinforce skills development. Online communities of practice and peer exchange systems can be useful but they need to be tailored to the health workers' needs, organized, and evaluated. If senior staff are not onsite, then at least monthly virtual or face-to-face meetings and case reviews by senior staff should be facilitated. Structured orientation and community-based projects for new staff can improve transition to new workers to rural work and support their interest in continuing the role.	How can rural workers be professionally supported? • What senior clinician support and supervision is available? • Are the information systems available to the health workers optimal for the job? • What systems (outreach, telehealth and onsite) are there for getting feedback on challenging cases? • What refresher courses and simulations could be available for low volume but important skills? • How can the health workers access peer support - professional meetings and practice discussions? • What professional networking is possible? • Are there opportunities to participate in local research projects? (21, 97, 106–128)
Monitoring and evaluation	Monitoring and evaluation of rural pathways plays a central role in informing any adjustment to the pathways as well as providing evidence about the effect on rural workforce, their supply, qualifications and retention, accessible health services and demonstrating community health, social, and economic outcomes. This requires consideration of routine data collection for pre and post testing or using control groups of rural communities without pathways.	Are the activities and outputs of the program being implemented as planned?What are the intended outcomes of pathways and how can we collect data to measure this effect? • Do we have workforce registries and health data or how can these be built and managed? • Are partnerships set up for strong evaluation? • What do we want to measure? - Is community need being monitored? - Is selection and training effective for pathways goals? - Are there more rural students / local workers and supervisors? - Is professional development effective?
		- Is there more infrastructure? - Is workforce retention better? - Is access to health services better? (earlier intervention, continuity and prevention measures) - Have there been changes in service volume and complexity? - What are the social, economic and health outcomes in the community? (7, 21, 39, 129, 130)

a *Please see the graphically-designed Checklist in Data Sheet 1*.

### Consultation

The consultation with the Expert Reference Group at round 2 (Table 2), suggested that the draft Checklist was well-structured and comprehensive—applicable to any LMICs, stakeholder, health worker, context, or health problem, regardless of the starting point. The reflective questions were considered useful:

*“In resource poor settings, a checklist of basic tools/equipment for the workers being trained is an important practical aspect for reflection”*.

*“Reflective questions fit our setting which is poorly resourced (human and material)”*.

The stakeholders gave feedback that they preferred that the evidence was provided in summary references only. Further, some respondents indicated that the Checklist may be more successful if there was more consideration as to the context of implementation. To address this, the Steering Committee decided it was relevant to develop a stakeholder map to accompany the Checklist, which could represent the partners in relation to implementing the 8 Checklist actions (Data Sheet 2). Also, a series of rural pathways implementation exemplars was developed. These were based on the highest quality examples of rural pathways activities that had been identified by the policy, scoping review and consultation phases, targeting coverage of different WHO regions (Data Sheet 3) (35, 131). Round 2 feedback confirmed that the term “*rural pathways*” was preferred over terms like “*rural pipelines*.” Respondents noted the former was easier to translate in all LMIC languages and encompassed different entry points, iterative lifelong learning and progression through training and development events, rather sealed and inflexible routes.

The round 3 feedback from the Expert Reference Group related to the graphically-designed version of the Checklist (131) (Data Sheet 1). This unanimously confirmed that the Checklist was comprehensive and complete:

“It's very well structured and is the way forward for any country”

“This graphic display presents the key elements very clearly”

“…I think it can be used at many different levels of the healthcare system and planning processes… to identify gaps.”

This feedback also identified that the priorities and the degree of progress with rural pathways implementation across the 8 actions was highly variable by country. Respondents reported that a key enabler for implementing actions were a government commitment to rural health, formalized in policy, and through sustained investment. They also mentioned that health service engagement and using coordinators who could assist to broker pathways and relevant partnerships may be helpful. The key barriers to implementation in LMICs included: time and funding; sharing resources with rural communities and; embedding the value of rural work and learning into policy action.

### Dissemination, Engagement, and Field-Testing

Dissemination, engagement, and field-testing provided a range of positive feedback about the Checklist and no major changes were suggested. Participants unanimously noted that the tool was highly applicable as a practical resource for implementing rural pathways in HICs (reaching beyond the intended purpose). The self-assessment tool (Data Sheet 4) was used to test the Checklist's application. Overall 56 participants from 20 countries participated in this process. Of these, 38 worked in rural areas, 21 were from LMICs—Brazil, Liberia, Guatemala, Malawi, India, Papua New Guinea, Latin America, Philippines and Uganda, and 35 were from HIC—Canada, Australia, United Kingdom, and United States of America (5 of whom were serving LMICs– Haiti, Kyrgyzstan, Syria, Iraq, Thailand, Asia, and Africa). Participants represented various stakeholders (director to student/community levels) from a range of organizations including universities, trainers/educators, global and rural health programs and researchers, overseas missions, policy and planning institutes, health services, and community boards.

Participants identified a wide range of real-world rural health problems and health workforce challenges for which the Checklist was considered relevant. The data from the self-assessment tool also showed that the Checklist was effective for any type of stakeholder to self-identify gaps in their rural pathway actions for specific rural health workers they were trying to train, develop, and support. Stakeholders suggested that it helped them to see gaps in the pathway that they needed to discuss with other stakeholders, so they could work out jointly agreed strategies to address these. Some considered that the Checklist and associated materials could be valuable to use with different rural pathways implementers in the one training sub-system, for example during collaborative meetings, to agree on community needs, stakeholder actions, and responsibilities, thereafter checking in regularly to refine action. The self-assessment tool was also considered applicable to support regular planning over the long-term rural pathways implementation cycle. This includes when reflexive action may be needed based on changing conditions. A summary participant's reflections is outlined (Table 4). Beyond the field-testing meetings, selected stakeholders applied the Checklist to real-world planning in their countries. One such study is published to date, finding the Checklist was applicable for developing strategies to expand rural healthcare workforce in Kyrgyzstan (132).

**Table 4:** Stakeholder feedback about the Checklist, following field-testing.

*The Checklist and self- assessment tool really helped me with my issue of developing online medical education*
*I identified lack of recognition as an issue for doing the training*
*It is adaptable to recruitment and retention in any population or health worker group*
*I was initially concerned but it relates significantly to me*
*It is exactly the type of tool I am looking for*
*I identified weaknesses in the project I am working on*
*It is possibly a resource for measuring outcomes of rural based training*
*I realize we need to decentralize care. It helped me map the new interventions we need*.
*I can take it to a group or practice community involved in developing a rural pathway to help them plan together and get a common agreement*
*Nothing helped me as closely to work out what we had to do than the Checklist*
*It could help us to appraise our new national training system*
*I knew the problem already but I realized I have not addressed all the issues so this helped me work out how effective the program is*
*It could be good to facilitate brainstorming and illuminate on the gaps because each part of the rural pathway is so complex, and some of them we know more than others*
*… could be used to check-in regularly with others involved in implementation, our perceptions may differ My answers about our progress would possibly be different to policy-makers*
*We get so many initiatives from top down and we get caught in expectations of being accountable to funding bodies that we forget to be accountable to the community. This tool reminded me about that*.
*This has application in building workforce capacity for primary care innovation, which will help us to keep people out of hospitals…tools like this can drive community prevention as well*
*It could help to bring entities together to find a common purpose*
*…It makes things explicit, brings them up for discussion*.
*…We are glad the Checklist fits the whole workforce and any problem… not just country level stuff but also local situations*.
*…helped me see the gaps. We may identify cost-neutral strategies from some of the prompts*.

With respect to HIC use, stakeholders noted that the state of progress toward rural pathways implementation was fairly strong, but the Checklist particularly applied to them as they noted their rural pathways were complicated by extensive numbers of stakeholders, professional competition, and workforce regulations which sometimes detracted from addressing community goals.

## Discussion

This project involved developing an agreed terminology and framework to support practical implementation of rural pathways in LMICs. The resulting Rural Pathways Checklist evolved from a breadth of methods including reviewing evidence, consultation, and engagement and field-testing to ensure it was both evidence-based and pragmatic, for greatest utility in LMICs. The Checklist is a step forward in achieving a globally-agreed conceptual framework and language which integrates eight training and personal/professional support actions to train, develop, and support rural health workers under a continuous quality improvement cycle. Building on the 2010 WHO recommendations for increasing access to health workers, the Checklist actions are focused on a community goal. They attempt to drive more comprehensive rural pathways interventions through long-term effort of many stakeholders, rather than through discrete or siloed strategies. We note from our consultation, that for LMICs to achieve holistic rural pathways implementation, relies on political commitment to decentralize training and resources to rural communities over the long-term. The Checklist may be a tool that LMICs can use to advocate for clear rural training/professional support strategies and more rural investment.

Developed with LMICs evidence as the reference point, the Checklist is the first of its type to be sensitive to countries with the most extreme healthcare needs and most significant shortages of health workers. Although HICs found the Checklist useful and applicable to their own setting, its application may have the most benefit in LMICs given the outstanding levels of socio-economic and health disadvantage, extreme geographical isolation, and poverty. Starting the process of implementing rural pathways in this context has the greatest potential to alleviate global poverty by increasing access to rural work and health, enabling universal health coverage. Critically, it may also bolster local health workers trained for the problems in the community who are more likely to be retained in their community of origin. By increasing trained health workers, local health services are able to cope with the volume of presentations locally and diversify their service platform, supporting rural social, and economic development goals. The demands for a skilled and stable rural health workforce have been particularly notable during the COVID-19 pandemic. The pandemic situation has placed extensive strain on a small number of rural health workers to work at a broad scope, often without the training and professional support they may need. The Checklist may be a way to navigate out of this situation and build self-reliant rural communities with sufficient skilled health workers.

The project identified that countries, districts, and communities are at different starting points with respect to rural pathways implementation and have different health needs, but the Checklist may be a viable reference point for planning and evaluating action regardless. As opposed to other tools that are discipline, stakeholder or country specific, the Checklist may aid as a resource that is applicable to diverse countries, rural locations, and workforce issues and help to break down siloed profession-based workforce planning, in vertical disease areas. Such effort does not address holistic rural community need in LMICs. The self-assessment tool provides a means of regularly evaluating progress with rural pathways implementation in the complex dynamic environment of rural communities, where changes are common and adjustment is regularly needed. Regular use of the self-assessment tool may increase the potential for timely identification and response to emerging challenges.

Our project has some limitations. The scoping review identified 127 relevant studies from LMICs, applicable to the Checklist but as more evidence emerges, the Checklist may need to be updated. We only commenced the process of collecting exemplars from different WHO regions in this project. Yet there is great potential to build on this and foster global communities of practice in this area, including through online exchanges. This may help with information-sharing and mitigate professional isolation that many rural pathways implementers may face in their own countries or regions. The Checklist has the potential to support more comparative multi-national research about rural pathways. Its utility may also be facilitated if further work was done to marry this with a suite of other global tools to guide action for improving universal health coverage worldwide. The field-testing we did was limited to a convenience sample covering diverse rural contexts, but further research could usefully test the applicability of the Checklist to a set of rural pathways stakeholders of individual countries such as some studies are starting to do (132). Finally, our research explored whether the Checklist applied to all types of health workers needed in rural areas, but it may be pertinent to test how well it applies to particular primary health cadres across a sub-set of LMICs, to gather more data about its reliability.

## Conclusion

Our study developed a Rural Pathways Checklist to support practical implementation of integrated rural training, development, and support strategies for health workers in LMICs rural areas. Through diverse methods which drew on both theory and practice, we identified eight actions, reflective questions, and additional resources. Together, these support a continuous, connected and sustained effort for rural pathways implementation in LMICs. Although this Checklist requires further validation, it is possible that it can produce real improvements in access to health workers, fit for the needs of LMICs communities worldwide, as a key step for addressing major inequalities in rural health and sustainable development in rural places.

## Data Availability Statement

The raw data supporting the conclusions of this article will be made available by the authors, without undue reservation.

## Ethics Statement

The studies involving human participants were reviewed and approved by This study had ethics approval from Monash University, Victoria, Australia (Project Number 17636) ratified by the University of Queensland (Project Number 2019002437). Written informed consent for participation was not required for this study in accordance with the national legislation and the institutional requirements.

## Author Contributions

BO'S led the conceptual design, ethics, data collection, field-testing, analysis, and writing. BC and JW-J led the establishment of the global and expert steering groups and the concept testing phase. AB assisted with the scoping review, expert reference group surveys, and field-testing protocol. All authors contributed to design, data collection, analysis, and writing and agreed on the final manuscript and Checklist materials for publication. The WHO approved the Checklist and final manuscript for submission for peer review publication.

## Conflict of Interest

The authors declare that the research was conducted in the absence of any commercial or financial relationships that could be construed as a potential conflict of interest.

## Supplementary Material

Data Sheet 1 Graphically-designed Checklist.

Data Sheet 2 Stakeholder map.

Data Sheet 3 Exemplars from WHO regions.

Data Sheet 4 Self-assessment tool.
