# Supplementary material for: A Checklist for Implementing Rural Pathways to Train, Develop and Support Health Workers in Low and Middle-Income Countries
Source: Front Med (Lausanne). 2020 Nov 27;7:594728. doi: 10.3389/fmed.2020.594728 (PMC7729061; doi:10.3389/fmed.2020.594728)
Supplement: Data Sheet 1 — Graphically-designed Checklist. [file Data_Sheet_1.PDF]

A Checklist for implementing rural pathways to train and support health workers in low and middle income countries

Adopting the rural pathways approach

This document presents an evidence–informed Checklist for implementing rural pathways to train and support the rural health workforce in low and middle income countries (LMIC). Rural areas are the most underserviced around the world. Governments, NGOs, health services and communities have attempted to address health workforce supply by using single solutions. In contrast rural workforce interventions are now recognised to require multi–dimensional approaches. This Checklist consolidates the parts needed for a more holistic and coordinated system of teaching, training and supporting the rural workforce in LMIC.

A Checklist for implementing rural pathways

This Checklist has been developed by the Wonca Working Party on Rural Practice in collaboration with Monash University based on a review of LMIC literature and policies. Many global stakeholders provided input during a global consultation process to shape and fashion the document. It is a Checklist for developing all types of skilled and qualified rural health workers, so as to build strong primary care teams working at broad scope to address the needs of rural communities.

It includes eight inter–dependent action areas, each of which is important. For each of these, a series of reflective questions and a summary of evidence is provided. We found a wide range of literature and strong exemplars about rural training approaches in LMIC which which provide a culturally and contextually rich perspective of the implementation context. Evidence confirms that the more actions from the Checklist which are implemented, the greater the effect of the rural pathway for achieving the rural workforce that is needed.

As a resource, the eight action areas of the Checklist will allow governments, educators, health services, researchers and community stakeholders to reflect on their current rural pathway activity and plan for more. The Checklist provides a framework for discussion and priority setting at country, district or community levels that will facilitate more comprehensive approaches to be achieved. At the centre of both priority setting and action, is the community. Developing rural workers in rural communities, for rural communities, on rural pathways, builds the health, social and economic outcomes of these communities.

Monitoring and evaluation is essential to ensure we build a training system which incorporates continuous quality improvement processes.

Rural pathways – putting stepping stones in place and then building

Consultation identified that rural pathways are enabled by clear agreement about pathway goals, community engagement, sustained partnerships and co–investment. Some aspects of rural pathways do not need funding, but can be achieved immediately through leadership and commitment. For other aspects, funding arrangements need to be discussed. One of the greatest investments is in funding dedicated leaders who are able to span all the sectors involved and support coordinated, sustained effort for rural pathways implementation. National and rural health policies are essential to build momentum and enable rural pathway action.

Rural pathways do not need to be of perfect tarmac before they can be traversed. Rural pathways can be constructed as a series of tracks with stepping stones and key bridges which can be built up by putting in constructively aligned components over time. Once started, it is possible to add a few gates, optional routes, destinations and further experiences. But as a starting point, even rudimentary pathways established around common goals and fostered by partnerships and co–investment, have every chance of succeeding. In the medium–term developing more comprehensive rural pathways will help to address the social, economic and health outcomes, for enormous gains in LMIC.

A Checklist – implementing rural pathways to train and support health workers in low and middle income countries

Checklist actions

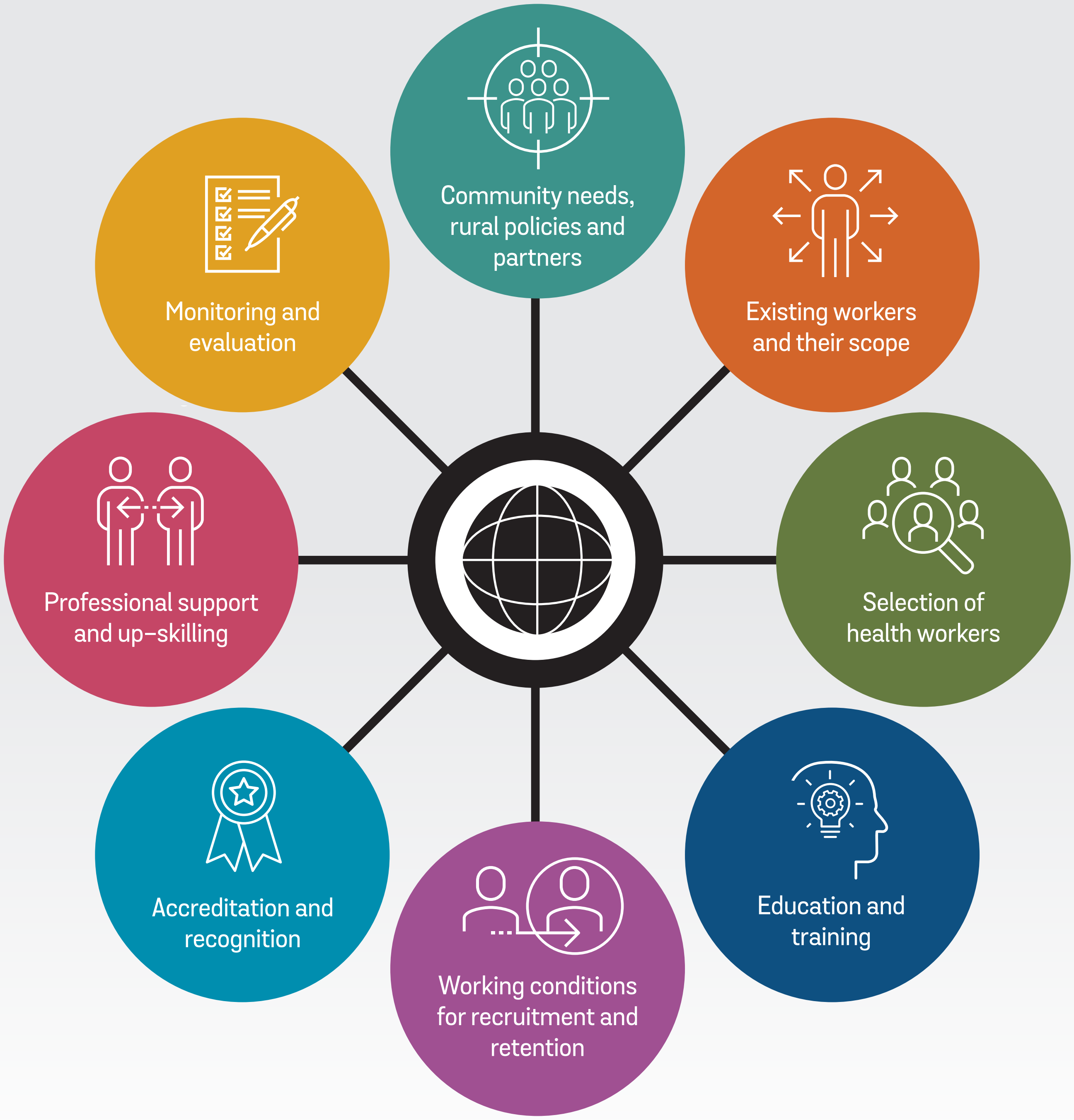

A Checklist – implementing rural pathways to train and support health workers in low and middle income countries

Community needs, rural policies and partners

Questions ?

What do our rural communities need?

- Is the community involved in defining priorities & solutions?
- Which of the needs can be addressed now, to build on later?

What rural health policies/plans exist to support action?

- Are they implemented?
  - Are they appropriately decentralised for local action?
- Are new policies needed?

What global, national or local partnerships could help?

- Are there partners who can assist?
  - How can partnership be sustained?

Evidence[a] ✓

First and foremost, working with rural communities is essential to define priorities and involve them in solutions. A scan of the national policies and plans for rural health provides insights into directions for governments and potential synergies between policies and the local priorities. Priorities for rural pathways may need to be sorted into an order, particularly in the face of competing demands for resources and in some cases, extensive unmet need. Government and other partners, along with decentralised finance and management is important for enabling solutions to be appropriately tailored and for ensuring appropriate technical and financial support is available.

Existing workers and their scope

Questions ?

What rural healthcare teams, working within what scope, are needed?

Do we already have workers with the skills for this scope of work?

- How have they been trained and where?
- What are their skills and qualifications/training for the demands of the role?
- Are they motivated to work at the required scope?
- Are they easily recruited/retained?
- Are there short-term recruitment options whilst longer-term workforce is developed?

Evidence[a] ✓

The current skill levels of rural workers may not be sufficient to meet rural and remote community needs. A scan of existing rural and remote health workers and their skills, practices and motivations can inform a clear rural pathways strategy. Rural and remote healthcare teams with a wider range of skills, supported by their employers, can improve comprehensive local care and potentially help to improve health worker satisfaction and retention. However, rural communities also need to balance short-term recruitment needs with long-term workforce building processes.

A Checklist – implementing rural pathways to train and support health workers in low and middle income countries

Selection of health workers

Questions ?

How can we select workers for this role from the community?

- Are there people in the rural community that could fill roles with some education and training?
- What process and criteria will effectively select them from the community for the community?
- What entry-level standard is appropriate for coping with the training?
- What financial and social support would make it easier for them to access education and training?
- What are the cost-benefits of training these people and how can the costs of training be shared?

Evidence[a] ✔

An extensive range of community selection options are demonstrated involving selecting people with a connection to "place", a commitment to serve others, and who are motivated to learn and invested in improving access to community health services.

Universities and training courses with a social accountability for developing health workers with a desire to serve others, trained and ready to work where they are most needed tend to have more tailored selection processes which improve overall outcomes. Selection of rural background people of different race and language groups relative to the country and rural context is important, along with financial and social support for these groups to fully participate in training courses. Cost-benefits of developing new workers are important considerations and should be evaluated.

Education and training

Questions ?

How can we effectively educate and train people in rural areas and for the breadth of skills needed by rural communities?

- What bridging courses are required?
- What rural curriculum is relevant? Who will develop and validate this?
- How can theoretical and practical components of training be delivered in rural areas?
- How much real-time face to face and virtual supervision will help people to learn practical skills safely?
- How can practical learning be structured to develop the scope and complexity of skills required?
- How much would it cost to train/employ/support trainees and how can this be funded?
- What further training and career options could be developed to enable qualified workers to keep progressing after the training?
- How can the local government, community and champions support the training?

Evidence[a] ✔

Optimal education and training for rural practice occurs through exposure to rural and remote practice, teams and health systems. Learning the range of skills needed is effective through distributed training systems using locally-available qualified teachers and supervisors, in the place where people are going to practice and involving of the people that the workers are going to help after they finish training. This often occurs within university and other training organisations with a social accountability for developing health workers from rural areas, who have a desire to serve others. Beyond any one course, there should be options for doing more advanced training for career progression. Training needs to cover the breadth of skills needed for the role. Sustainable funding and technical support for decentralised training is important.

A Checklist – implementing rural pathways to train and support health workers in low and middle income countries

Working conditions for recruitment and retention

**Questions** ?	How can we ensure practice conditions in the community promote health worker satisfaction, recruitment and retention?<br>• Are we recruiting people who completed training in the community to work in the community?<br>• Do the rosters make the workload sustainable?<br>• Are we creating jobs with satisfactory employment terms and variety, volume and scope of work?<br>• Is remuneration appropriately rewarding employees?<br>• Is there an orientation to the workplace?<br>• Is there orientation to the community?<br>• Are senior workers and supervisors available onsite/virtual?<br>• Is there training for health service managers?<br>• What support is there for housing and meals?<br>• Do health workers have transport for their work?<br>• Are there baseline stocks of medical supplies, equipment and drugs?<br>• Are the health service buildings and clinical infrastructure of reasonable standard?<br>• Is there security for workers?<br>• Are workers given enough time off?<br>• Are there subsidies for work away from home?<br>• Do workers have access to technology support and internet?<br>• Is there rural health team cohesion?
**Evidence**[a] ✓	Education and training is only likely to be effective in recruiting and retaining health workers if the practice conditions are right, there is a supportive learning culture in the health service, there are sufficient supplies and clinical infrastructure, good remuneration, and sustainable workload. Health worker motivation and engagement is better if employers regularly check in with them about their goals and any factors impacting their performance. Structured orientation and community-based projects for new staff can improve transition to rural work as a new worker and interest in continuing in the role.

Accreditation and Recognition

**Questions** ?	How can trained rural workers be accredited and recognised for transferability of the qualification?<br>• What qualification can they be given?<br>• How can the community value graduates of the training?<br>• Is there a professional title for graduates?<br>• Are the graduates recognised at country level for what they do?<br>• Can the graduates be paid appropriately for using the skills they have developed?<br>• Do they have options for progressing their career path?
**Evidence**[a] ✓	Accreditation and formal professional recognition of the worker is important for recognising their training and scope of work. It helps reinforce the value of their personal commitment in doing more training and supports their retention in the role and use of all their skills. It also helps the community to identify qualified health workers.

A Checklist – implementing rural pathways to train and support health workers in low and middle income countries

Professional support and up-skilling

**Questions** ?	How can rural workers be professionally supported?<br>• What senior clinician support and supervision is available?<br>• Are the information systems available to the health workers optimal for the job?<br>• What systems (outreach, telehealth and onsite) are there for getting feedback on challenging cases?<br>• What refresher courses and simulations could be available for low volume but important skills?<br>• How can the health workers access peer support – professional meetings and practice discussions?<br>• What professional networking is possible?<br>• Are there opportunities to participate in local research projects?
**Evidence**[a] ✓	It is important to provide professional supervision and networking opportunities to reduce health worker isolation and reinforce skills development. Online communities of practice and peer exchange systems can be useful but they need to be tailored to the health workers' needs, organised and evaluated. If senior staff are not onsite, then at least monthly virtual or face to face meetings and case reviews by senior staff should be facilitated.

Monitoring and evaluation

**Questions** ?	Are the activities and outputs of the programme being implemented as planned?<br>What are the intended outcomes of rural pathways and how can we collect data to measure this effect?<br>• Do we have workforce registries and health data or how can these be built and managed?<br>• Are partnerships set up for strong evaluation?<br>• What do we want to measure?<br>– Is community need being monitored?<br>– Are selection and training effective for pathways goals?<br>– Are there more rural students/local workers and supervisors?<br>– Is professional development effective?<br>– Is there more infrastructure?<br>– Is workforce retention better?<br>– Are health services of higher quality? (earlier intervention, continuity and prevention measures)<br>– Have there been changes in service volume and complexity?<br>– What are the social, economic and health outcomes in the community?
**Evidence**[a] ✓	Monitoring and evaluation of rural pathways plays a central role in informing any adjustment to the pathway as well as providing evidence about the effect on rural workforce supply, qualifications and retention. Effects will also be expected in accessibility of health services and improved community health, social and economic outcomes. This requires consideration of routine data collection for pre and post testing or using control groups of rural communities without pathways.

[a] Evidence is based on a scoping review of 127 articles identified in relation to the rural training pathways for the health workforce in low and middle income countries 1998–2018, a global consultation and review of global human resource for health policies.
