# Supplementary material for: A Checklist for Implementing Rural Pathways to Train, Develop and Support Health Workers in Low and Middle-Income Countries
Source: Front Med (Lausanne). 2020 Nov 27;7:594728. doi: 10.3389/fmed.2020.594728 (PMC7729061; doi:10.3389/fmed.2020.594728)

Applying the Checklist to implement a rural pathways approach

Who is involved and how?

Rural pathways to train and support the rural health workforce involve an integrated system of measures and stakeholders. To produce effective outcomes, all the elements need to be synergistic and coordinated. Without key elements they can be dysfunctional or destructive.

At the centre, as the main key to driving the system, is the rural community or the region. Communities who are engaged in selecting, developing, supporting and monitoring their own rural health workforces are essential for rural pathways to be appropriately tailored, sustainable and effective. The diagram depicts the community as the cogwheel about which any other action for implementing rural pathways revolves.

All of the other elements of the checklist actions, represented in the other wheels are required to support the process and also articulate with the community. They are fundamental to the progress of the pathway. The actions in the areas of selection, education and training, professional development and upskilling, worker support and meaningful qualifications and recognition are intrinsically linked with the need for monitoring, evaluation and quality improvement.

For these actions to occur, an integrated and sustainable layer of partnership between stakeholders, committed to the workforce needs of rural communities needs to occur. This layer wraps around and invests in rural training pathways as a seamless belt around the whole system. The tread of this layer is broad enough to buffer the wheels, including the community, from external forces. It holds the actions and the community together and harnesses the power of the community. With all of these components interplaying, synergy and synchrony is possible and sustained progress in rural health workforce training pathways occurs.

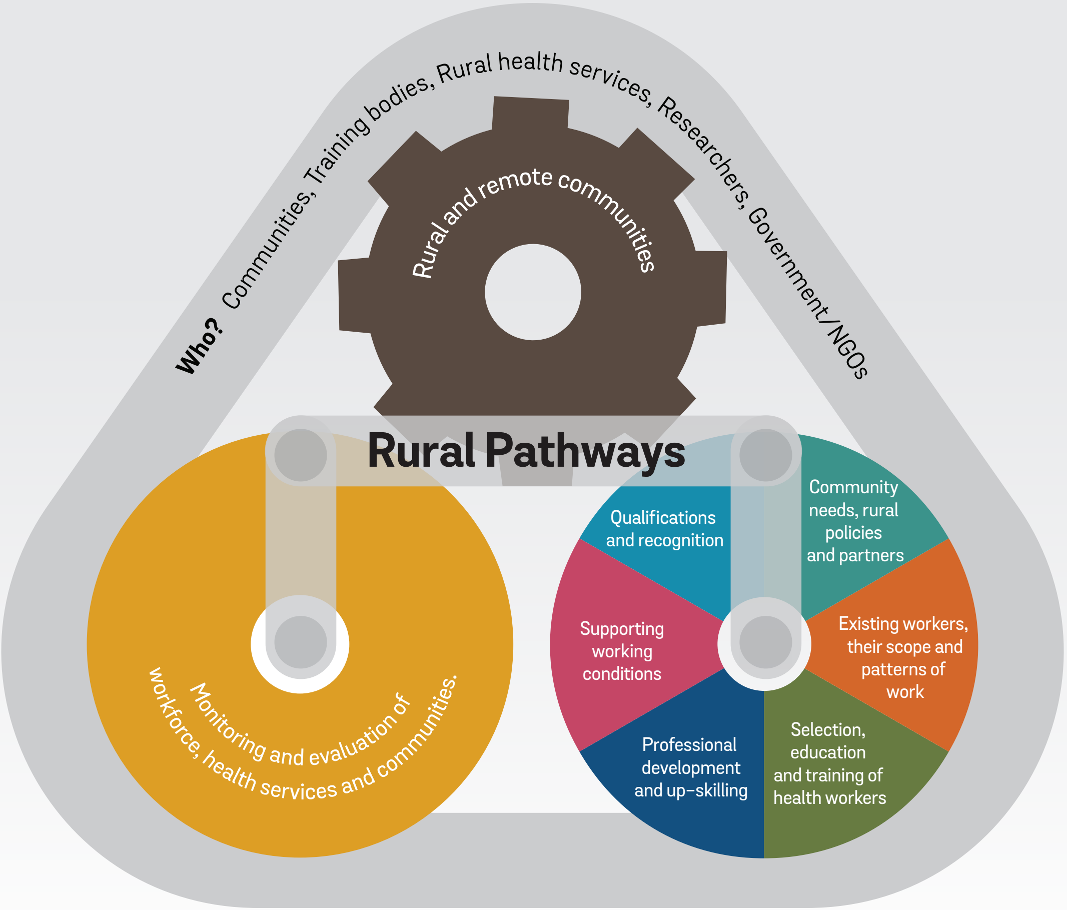

Supplement: Data Sheet 2 — Stakeholder map. [file Data_Sheet_2.PDF]
