# Supplementary material for: A Checklist for Implementing Rural Pathways to Train, Develop and Support Health Workers in Low and Middle-Income Countries
Source: Front Med (Lausanne). 2020 Nov 27;7:594728. doi: 10.3389/fmed.2020.594728 (PMC7729061; doi:10.3389/fmed.2020.594728)
Supplement: Data Sheet 3 — Exemplars from WHO regions. [file Data_Sheet_3.PDF]

Applying the Checklist to implement a rural pathways approach

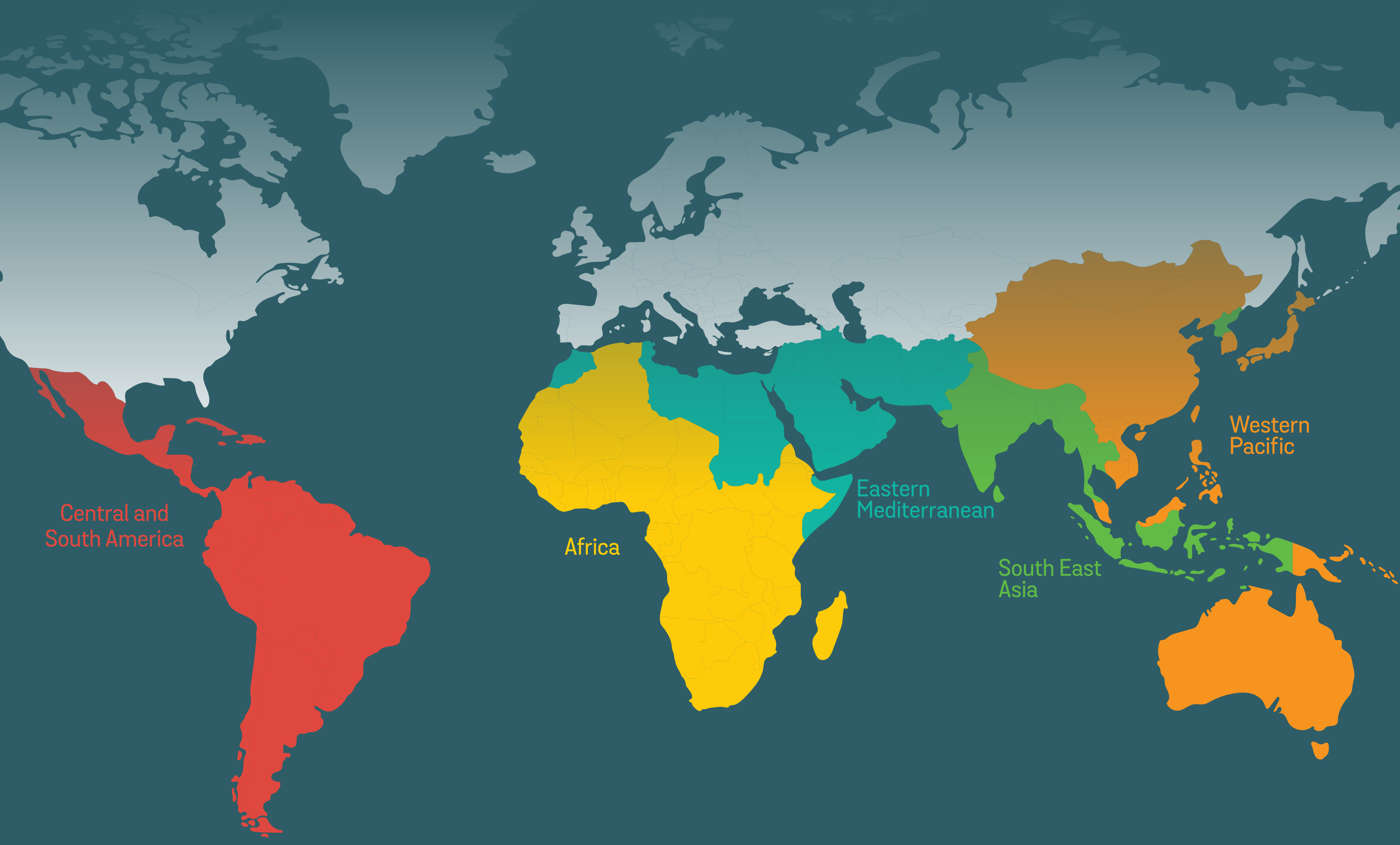

Where can we find exemplars in our region?

The Checklist for implementing rural pathways outlines the component actions for integrated rural pathways to train and support of the rural health workforce in LMIC.

Implementation of the Checklist actions can be informed by examples. The following exemplars have been drawn from a range of disciplines, LMIC countries and WHO regions. Together they span the globe and provide some insights into effective approaches that could be adopted. Many may also support the development of new and effective collaborations.

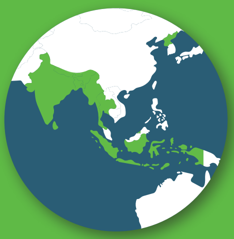

South East Asia

EXEMPLARS[a]	IMPLEMENTATION
**The Collaborative Project to Increase Production of Rural Doctors (CPIRD)**	The Collaborative Project to Increase Production of Rural Doctors (CPIRD) was initiated in 1994 in Thailand as a policy targeting rural selection into medical school and rural training tracks with rural return of service as an innovative example of educational policy in a LMIC. Since 2005, the CPIRD has initiated 4 different tracks (regular CPIRD) to select rural high school students, one district-one doctor programme (ODOD) (for remote high school students), a regular post-graduate programme (for people with medical-related bachelor degree) and a post-graduate programme for civil servants with medical-related bachelor degrees. It has been comprehensively evaluated using control groups to show positive academic, rural recruitment and retention results.
**Fellowship in Secondary Hospital Medicine (FSHM) by Christian Medical College (CMC) Vellore**	In India a Fellowship in Secondary Hospital Medicine (FSHM) is a year-long blended on-site and distance learning programme implemented in 2007 by Christian Medical College (VMC) Vellore. It provides education and professional support for junior doctors doing required rural service terms in rural hospitals. It consists of 15 paper-based distance learning modules to support skills for work in rural hospitals, professional networking, and project work focused on improving rural services with promising results for participants.
**National Rural Health Mission in India develops Social Health Activists**	The National Rural Health Mission (NRHM) started in India in 2005, focused on 18 deprived rural states to increase access to institutional antenatal care. Decentralised funding and planning assisted states to define their health priorities. Training was expanded for developing accredited Social Health Activists (ASHA) in partnership with the non-government sector. These were female workers drawn from the same community they intend to work in. Evaluation showed an impact on health and social inequalities in high focus NRHM states, with increased uptake of maternal healthcare, and decline in socioeconomic inequity.
**Christian Medical College (CMC) Vellore provides decentralised general practice training**	To improve the skills and confidence of rural doctors to manage the wide range of conditions they see in the community, the CMC Vellore launched an initiative to "refer less resolve more". It offers a "two year family medicine diploma course" for rural doctors by distance mode. It is an innovatively-written programme consisting of problem-based self-learning modules, video-lectures, video-conferencing, and face-to-face contact programmes. Ten secondary level hospitals across the country, under the supervision of national and international family medicine faculty, form the pillars of this programme. Between 2006 and 2011, 942 general practitioners were enrolled.
**A government of Nepal and Nick Simons Institute (NGO) partnership for training family physicians**	In 2006 the government of Nepal and Nick Simons institute (NGO) progressively implemented a programme to develop family physicians for remote Nepalese hospitals. Firstly, 1-2 qualified family practice doctors were recruited per programme and hospital. Students were competitively selected and given scholarship support and bundled incentives for participating in the three year post-graduate programme with a binding contract to work in the remote hospital for three years. In-service training was provided for all staff for an effective hospital team. All seven programme hospitals became providers of emergency obstetric care and doctors did 10-50 caesarian sections per year and lessons learnt were the need for continued refinement of the pathway for addressing emerging issues.
**Family Medicine Programme (FMP) in Timor-Leste**	In the year 2000, there were approximately thirty Timorese doctors in Timor-Leste. Today there are almost 1000. The majority of those doctors have been trained either at the Escuela Latinamerica de Medicina (ELAM) in Cuba, or via an ELAM-supported programme at the Universidade Nacional Timor Lorosa'e (UNTL). Under the Gusmao government's 'Doctors for the Districts' policy, new graduates were being sent out to live and work in rural communities across Timor-Leste, until it was realised that these young doctors lacked the knowledge and skills necessary to provide safe, quality primary care in these contexts. Thus, in 2014, the inaugural Family Medicine Programme (FMP) was established. This initiative involved a collaboration between the Ministry of Health (MoH), UNTL and the Royal Australasian College of Surgeons (RACS), who subsequently requested technical support from Rocketship Pacific Ltd (Rocketship) – an international health charity dedicated to strengthening primary healthcare systems in Pacific island countries. Rocketship supported the design and delivery of the Family Medicine Programme. Using the Primary Curriculum of the Australian College of Rural and Remote Medicine (ACRRM) as its basis, the FMP is in its fourth year in 2018.

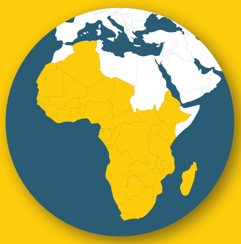

Africa

EXEMPLARS[a]	IMPLEMENTATION
**Walter Sisulu University Faculty of Health Sciences (WSUFHS)**	The Walter Sisulu University Faculty of Health Sciences (WSUFHS), formerly the University of Transkei Medical School, was founded in 1985, with a traditional curriculum. It was re-engineered in1992 to focus on community-based, socially accountable medicine in order to train physicians capable of providing quality health care in rural South African communities, particularly in the Transkei region. Instead of academic record, students are selected with 60% pass in mathematics and physical science and by demonstrating good thinking, communication and interpersonal skills and motivations. It has enrolled: 430 (83%) black Africans; 68 (14%) Asian descendants; 8 (2%) mixed race; and 5 (1%) whites with good retention of African students (<10% drop out).
**Clinical Associates training in South Africa**	South Africa introduced a new cadre of Mid-Level health worker in 2008, the Clinical Associate, to address the skills gap in district hospitals and three institutions started training these professionals selected as young people from socially disadvantaged communities. Between 2008 and 2017, 937 Clinical Associates graduated and of these, 80% serve in rural public health services. The cost of training a Clinical Associate was noted to be less than half that of training a medical practitioner and 2.4 clinical associates can be employed for the cost of one physician. The clinical associates provide essential capacity for health services, especially to those with the highest need – the rural and urban poor.
**University of Nairobi decentralised medical education**	University of Nairobi increased the number of medical students three-fold to produce more doctors for the population's needs supported through a Medical Education Partnership Initiative (MEPI) programme (a collaboration with the University of Washington, University of Maryland). It started training medical students in decentralised hospitals in 2011. Training was for 4th year students of 5 year course, including training and supporting preceptors. The training was 7-weeks long, done in small groups (3 per consultant) and included interactive learning as a group, through online webcast weekly. The rural experience is only short but students enjoyed this.
**A partnership in Tanzania for training Community Health Workers**	In Tanzania, a Connect project partnership between Ifakara Health Institute (IHI), Tanzanian Ministry of Health and Social Welfare (MoHSW), Tanzanian Training Centre for international Health (TTCIH) and Columbia University's Mailman School of Public Health (CU-MSPH) was launched in 2011. The project provides funds to Kilombero, Ulanga and Rufiji districts for human resources and supplies. Community Health Workers are selected from their communities with minimum skills for 9 months training and then redeployed home under supervision of trainers and a local clinical officer. Workers were given equipment (bicycle, mobile phone, and clinical infrastructure) and the community are required to employ the health workers including salaries and social security benefits. By August 2012, 113 community health workers were trained in 50 intervention villages at a cost of US$ 2,489.30 per health worker training including 40% for meals and 20% for accommodation and 8% for training and 10% for tuition fees.
**A framework for decentralised health worker training in South Africa**	In South Africa a collaborative co-design project, the Stellenbosch University Collaborative Capacity Enhancement through Engagement with Districts (SUCCEED) worked with representatives of academic institutions, government and health services as well as with a range of health professionals to develop a framework for effective decentralised training for healthcare professionals. The vision for collaborative, distributed training across all service platforms, and particularly in rural areas, was captured in a consensus statement adopted by the South African Association of Health Educationalists (SAAHE) in 2017, which has been endorsed by many bodies across the country. The Framework is being used by a number of institutions to guide implementation of rural training.
**Scaling up mid-level worker and medical training in Ethiopia**	In 2003, the Democratic Republic of Ethiopia invested in the selection, training and recognition of primary health workers through the Health Extension Programme. By 2010, a total of about 34,000 HEWs were trained and deployed throughout the country and concurrently around 15,000 health posts were constructed in the country. Additional aims of a broader human resource strategy were to increase eligibility and uptake of training as mid-level workers for health centres and district hospitals and develop more medical doctors by 2015. Between 2003 and 2009, the number of universities and health science colleges grew from five to 23. The original five medical schools were asked to increase their annual enrolment by up to four times. A range of Medical Education Partnership Initiatives (MEPI) between Ethiopian Medical Schools and US Partners helped to ensure quality medical education and retention of doctors in rural areas throughout this period of rapid growth.

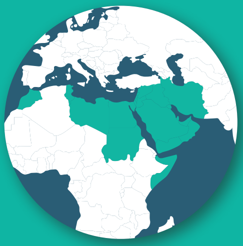

Eastern Mediterranean

EXEMPLARS[a]	IMPLEMENTATION
**National Community Health Worker Training in Iran**	National CHW training existed since 1979 in Iran and has been regularly reviewed. Workers are selected from their own community and employed in Village Health Houses in rural areas. The 2-year training is provided in 224 specialised centres. Then in-service training is provided at regular intervals by GPs and allied health workers in rural sites and village Health Houses. Nationally, 31,000 Community Health Workers (CHW) staff primary care units in these rural areas but the scope of practice required to address rural community need requires regular up-skilling.

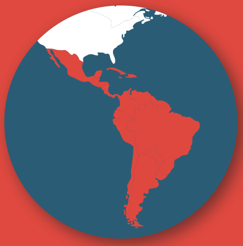

Central and South America

EXEMPLARS[a]	IMPLEMENTATION
**Mais Medicos programme in Brazil**	The Mais Medicos programme was a national policy implemented in Brazilian law in 2013. It had some innovative components such as increasing medical school enrolment, a new curriculum for medical schools and compulsory rural service, along with investment in health care infrastructure and improving Basic Health Care Units. By 2014, the MM provided an additional 14,462 physicians to highly vulnerable, remote areas in 3,785 municipalities (68% of the total) and 34 Special Indigenous Sanitary Districts and more than 50% physicians earned over 10 minimum monthly wages. But many enrolled in the programme were Cuban doctors and physicians refused to commit exclusively to one location and fail to comply with the stipulated working hours since they work under different contracts in several municipalities.
**Companeros en Salud (CES) – an education support package for junior doctors in rural service terms**	To provide a transformative learning experience for junior doctors doing their mandatory year of rural service, the Companeros en Salud (CES) aims to support them to deliver primary care, expose them to global health issues and engage them in socially accountable medicine. It also targets career development through supportive on site mentorship (from different USA based volunteer specialists and Mexican doctors) and supervision (from experienced clinicians), as well as monthly interactive seminars. The program has had good results for building medical knowledge, skills and orientation to serving the poor but some participants have had concerns about passing a residency entrance exam.

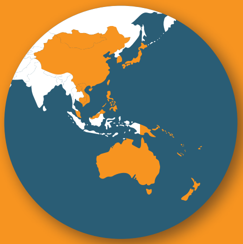

Western Pacific

EXEMPLARS[a]	IMPLEMENTATION
**University of Philippines Manila–School of Health Sciences (SHS–Palo)**	The University of Philippines Manila–School of Health Sciences (SHS–Palo) in Leyte was established in Eastern Visayas in 1976 with a social accountability mission to serve the region's poorest people, with isolated communities having the worst health outcomes. It selects students from lower socio-economic students from rural and remote communities, with scholarships for those nominated by rural communities who need health workers with social contracts to serve those communities at graduation. The students are selected based on "commitment to serve" not academic record. The curriculum is based on community needs. The school has options for graduates to move from community health work to a certificate in Community Health Work (Midwifery) to a Bachelor of Nursing (BSN) and on to a Doctor of Medicine.
**Ateneo de Zamboanga University (AZDU) School of Medicine**	Another regional medical programme emerged in the Philippines, established by the Ateneo de Zamboanga University (AZDU) School of Medicine. The AZDU programme was developed by local community leaders to serve the Western Mindanao region and to minimise students from the region having to leave home to study medicine in the city. It also had a strong focus on socially accountable medicine. The School has had a major positive impact on staffing public health services in the region.
**Master of Medicine (Rural) programme in Papua New Guinea**	A pioneering collaboration between the University of Papua New Guinea and the PNG Society of Rural and Remote Health led to the establishment of a Master of Medicine (MMed) Rural programme that has been running successfully for several years. This programme was developed in recognition of the need to provide a specially-selected cohort of postgraduate doctors with the knowledge and skills required to provide high-quality care in district hospitals across the mountains, tropical forests and remote islands of PNG. In addition to intensive training in the medical and procedural sub-specialties, MMed (Rural) graduates also work through health management disciplines such as finance and human resource management, as well as laboratory techniques, supply chain management and basic electrician training – all in a day's work for rural hospital doctors in PNG.
**Family Medicine training in Fiji and Tonga**	A new collaboration between Fiji National University (FNU), the Ministries of Health in Fiji and Tonga and Rocketship Pacific Ltd (Rocketship) – an international health charity dedicated to strengthening primary healthcare systems in Pacific island countries – is preparing to launch the South Pacific region's first-ever postgraduate family medicine training programme. Two cohorts will commence training towards their Diploma in 2019 – one in Fiji, the other in Tonga. The Tongan trainees will be supported by a team of experienced rural generalist medical educators recruited and deployed by Rocketship. This innovative model of training will allow the Tongan doctors to train towards their Diploma while living and working in their home hospitals and communities. The model is based on the great success of the Remote Vocational Training Scheme (RVTS) in Australia. It is hoped that new FNU programme will be expanded in subsequent years, to include other Pacific island countries, and be extended to Masters level.

[a] Evidence is based on a scoping review of 127 articles identified in relation to the rural training pathways for the health workforce in low and middle income countries 1998–2018, a global consultation and review of global human resource for health policies.
