# Supplementary material for: A Checklist for Implementing Rural Pathways to Train, Develop and Support Health Workers in Low and Middle-Income Countries
Source: Front Med (Lausanne). 2020 Nov 27;7:594728. doi: 10.3389/fmed.2020.594728 (PMC7729061; doi:10.3389/fmed.2020.594728)
Supplement: Data Sheet 4 — Self-assessment tool. [file Data_Sheet_4.PDF]

The University of Queensland
Australia
Create Change

Monash University

Wonca Rural Health
The Wonca Working Party on Rural Practice

Rural Pathways Checklist: Self–assessment tool

Checklist Actions

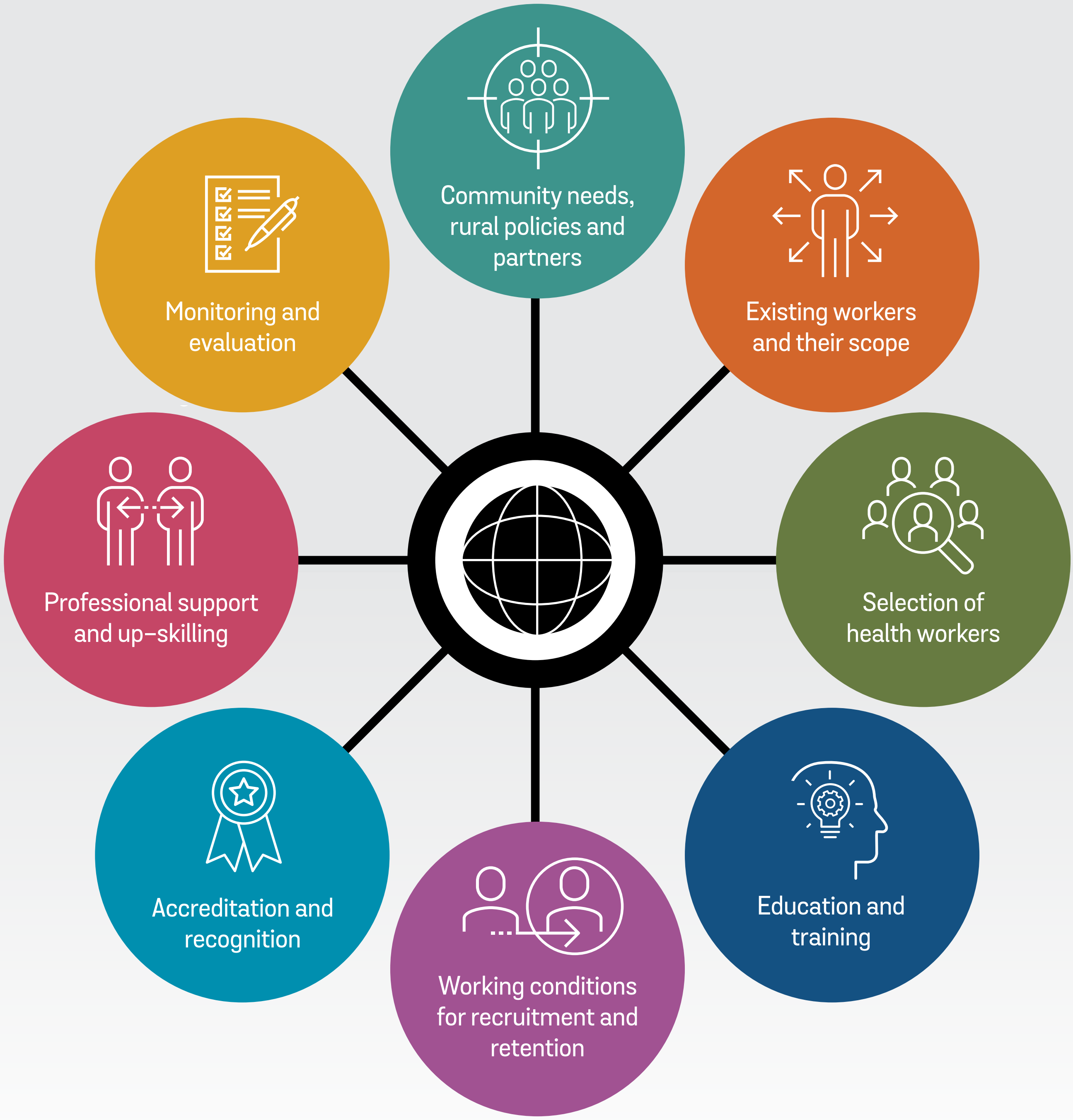

Rural Pathways Checklist: Self–assessment tool

This self–assessment tool aims to benchmark and plan the rural pathway for particular issues faced in rural communities

About you

**What is your name and title?**	
**What is your position?**	
**What is the name of your organization?**	
**Where is your office based? (town/suburb and country)**	
**Is this a rural location?**	
**Briefly describe your role**	
**Email**	
**Today's date**	

About your context

**What is/are the rural healthcare problem/s you are addressing?**
**Are there any particular health workers (and/or skills) that you need to address this and where?**

Rural Pathways Checklist: Self–assessment tool

Checklist Actions

For your problem area, please refer to the Checklist and complete the following questions.

Community needs, rural policies and partners

✓ Involving rural communities in defining priorities and potential solutions is important. Also national policies and plans for rural health play a strong role in supporting action.[a]

Please indicate progress from 0 to 4
0=not started
4=strong progress

**We are working with rural communities to define priorities and involve them in solutions for this problem**	
Discuss context, barriers and enablers:	
**We have scanned the national policies and plans for rural health and considered any new policies which would support us to implement a rural pathway for this problem**	
Discuss context, barriers and enablers:	
**We have got partners who can technically/financially support us to implement rural pathways to address this problem**	
Discuss context, barriers and enablers:	

**Score based on 12 available points**	***Pts:***	0	***%:***	0%

Existing workers and their scope

✓ Rural and remote healthcare teams who are appropriately trained and supported to use their breadth of skills improve access to comprehensive local healthcare. They are also more satisfied and likely to be retained.[a]

Please indicate progress from 0 to 4
0=not started
4=strong progress

**We have identified the skills and qualifications/training needed for the demands of the role**	
Discuss context, barriers and enablers:	
**We have identified the factors motivating and/or inhibiting workers in this area at the moment**	
Discuss context, barriers and enablers:	

**Score based on 8 available points**	***Pts:***	0	***%:***	0%

Rural Pathways Checklist: Self–assessment tool

Selection of health workers

✓ Selecting people with a connection to "place" and a commitment to serve others, motivated to learn and invested in improving access is important.[a]

Please indicate progress from 0 to 4
0=not started
4=strong progress

**We are selecting motivated people from the rural community who will be suitable for this role if they get education and training**	
Discuss context, barriers and enablers:	
**Our current selection process for these workers includes criteria about the person's connection and motivation for working in the areas we want to address**	
Discuss context, barriers and enablers:	
**We have decided an appropriate entry–level standard for the training**	
Discuss context, barriers and enablers:	
**We have considered financial support needed for helping rural people to participate in the training**	
Discuss context, barriers and enablers:	

**Score based on 16 available points**	**Pts:**	0	**%:**	0%

Rural Pathways Checklist: Self–assessment tool

Education and training

✔ Learning through distributed training systems using locally–available qualified teachers and supervisors, in the place where people are going to practice is important.[a]

Please indicate progress from 0 to 4
0=not started
4=strong progress

**There are relevant bridging courses and step–wise qualifications available for people in the rural community to access all levels of required training**	
Discuss context, barriers and enablers:	
**The curriculum includes rural practice elements for the scope and complexity of skills required in the rural community**	
Discuss context, barriers and enablers:	
**Relevant theoretical and practical components of the course are delivered in or near the rural areas where we want the workers to end up**	
Discuss context, barriers and enablers:	
**Real–time face to face and virtual supervision is available to support learning by rural trainees and workers in the areas we are addressing**	
Discuss context, barriers and enablers:	
**Local government, community and champions have been engaged to support the rural training**	
Discuss context, barriers and enablers:	

Score based on 20 available points	Pts:	0	%:	0%

Rural Pathways Checklist: Self–assessment tool

Working conditions for recruitment and retention

A supportive learning culture in the health service is essential along with sufficient supplies, clinical infrastructure, safety and sustainable workload.[a]

Please indicate progress from 0 to 4
0=not started
4=strong progress

**People who trained in the community can get jobs in the areas where they trained**	
Discuss context, barriers and enablers:	
**Trainees and workers are routinely orientated to the community and workplace when they arrive**	
Discuss context, barriers and enablers:	
**There are enough people on the rosters to make the workload sustainable**	
Discuss context, barriers and enablers:	
**Infrastructure and clinical resources are available in the area so that the trainees and workers can practice at the top of their skills**	
Discuss context, barriers and enablers:	
**We have put steps in place so that health workers feel safe**	
Discuss context, barriers and enablers:	
**We are giving the workers enough time off**	
Discuss context, barriers and enablers:	

**Score based on 24 available points**	**Pts:**	0	**%:**	0%

Rural Pathways Checklist: Self–assessment tool

Accreditation and Recognition

✓ Accreditation and formal professional recognition acknowledges the worker's training and scope of practice and reinforces the value of their personal commitment of doing more training.[a]

Please indicate progress from 0 to 4
0=not started
4=strong progress

**Graduates who complete the training are formally recognised by a qualification**	
Discuss context, barriers and enablers:	
**Graduates have a career path and can use the qualification in a range of settings, not just in the location/s where they trained**	
Discuss context, barriers and enablers:	
**People with qualifications are paid appropriately for being qualified to practice in the rural area**	
Discuss context, barriers and enablers:	

**Score based on 12 available points**	**Pts:**	0	**%:**	0%

Rural Pathways Checklist: Self–assessment tool

Professional support and up–skilling

✓ Professional supervision and networking opportunities (virtual or face–to–face) reduce health worker isolation and reinforce skills development.[a]

Please indicate progress from 0 to 4
0=not started
4=strong progress

**Senior clinician support and supervision is available for ongoing professional development by trainees and junior workers in the rural area**	
Discuss context, barriers and enablers:	
**Peer support, professional meetings and practice discussions are available and relevant for the trainees and workers**	
Discuss context, barriers and enablers:	
**Information systems are available for the trainees and workers in the rural area**	
Discuss context, barriers and enablers:	
**Rural workers can access refresher courses, workshops and/or research projects they may want to do**	
Discuss context, barriers and enablers:	

Score based on 16 available points	Pts:	0	%:	0%

Rural Pathways Checklist: Self-assessment tool

Monitoring and evaluation

Monitoring rural pathways allows for quality improvement and provides evidence about the effect on rural workforce supply, qualifications and retention along with the health, social and economic impacts for rural communities.[a]

Please indicate progress from 0 to 4
0=not started
4=strong progress

**The measures we will use to monitor the effect of training and support strategies have been identified**	
Discuss context, barriers and enablers:	
**We are collecting data to test the training and support strategies we are using**	
Discuss context, barriers and enablers:	
**We are reporting data about the implementation and outcomes of the strategies we used**	
Discuss context, barriers and enablers:	

**Score based on 12 available points**	***Pts:***	0	***%:***	0%

Rural Pathways Checklist: Self–assessment tool

Reflections

For a summary of your progress fill in the percentage score from the self–assessment action in the diagram below

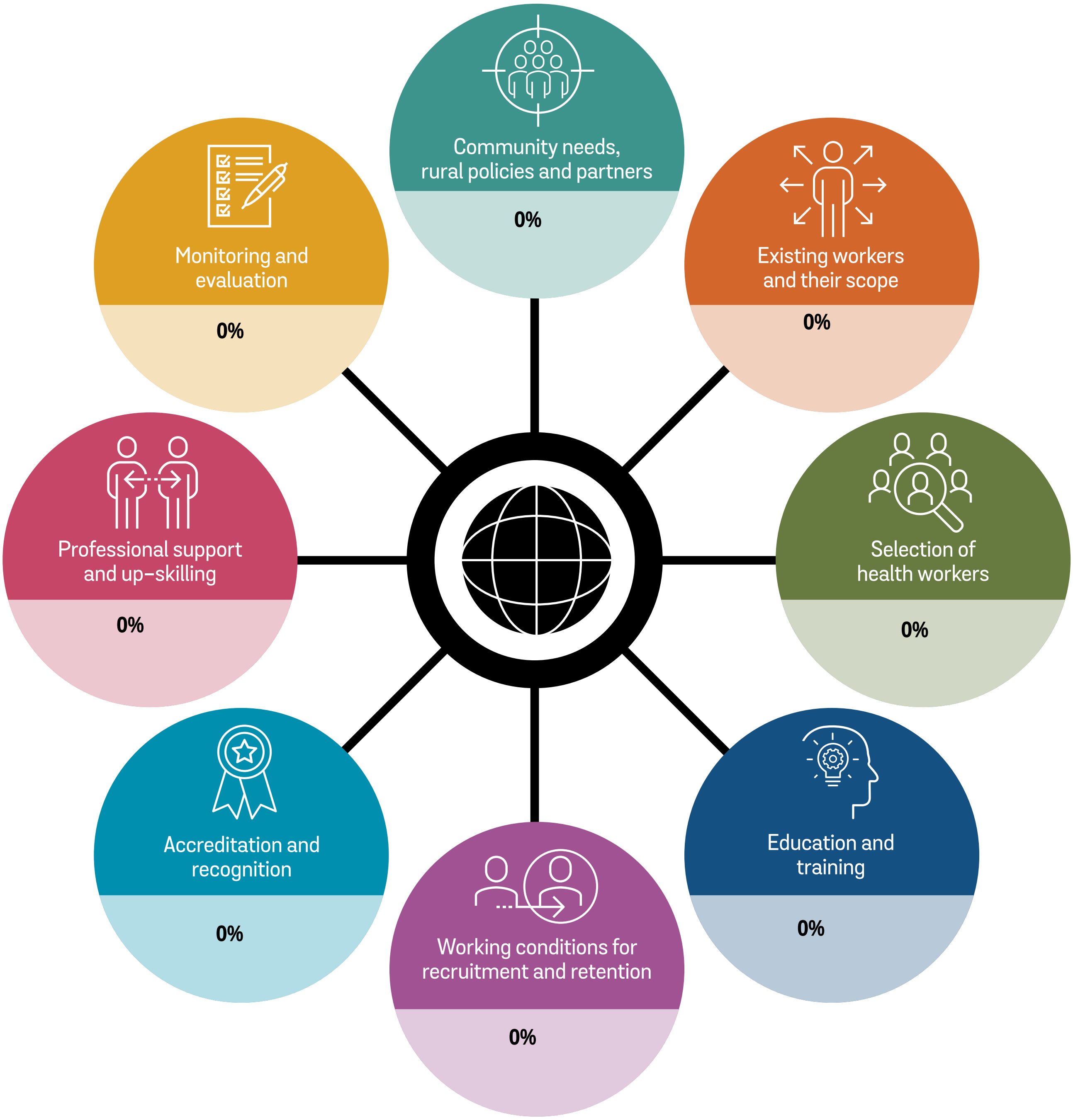

For more information please contact Dr Belinda O'Sullivan belinda.osullivan@uq.edu.au

[a] Evidence is based on a scoping review of 127 articles identified in relation to the rural training pathways for the health workforce in low and middle income countries 1998–2018, a global consultation and review of global human resource for health policies.

The Rural Pathways Checklist was funded by the WHO
